# Fear-Potentiated Startle and Fear Extinction in a Sample of Undergraduate Women Exposed to a Campus Mass Shooting

**DOI:** 10.3389/fpsyg.2016.02031

**Published:** 2017-01-06

**Authors:** Holly K. Orcutt, Susan M. Hannan, Antonia V. Seligowski, Tanja Jovanovic, Seth D. Norrholm, Kerry J. Ressler, Thomas McCanne

**Affiliations:** ^1^Department of Psychology, Northern Illinois University, DeKalbIL, USA; ^2^Department of Psychiatry and Behavioral Sciences, Emory University School of Medicine, AtlantaGA, USA; ^3^Trauma Recovery Program, Atlanta Veteran Affairs Medical Center, DecaturGA, USA; ^4^Howard Hughes Medical Institute, Chevy ChaseMD, USA; ^5^Program in Neuroscience, Department of Psychiatry, Harvard Medical School, BostonMA, USA

**Keywords:** posttraumatic stress symptoms, fear conditioning, fear-potentiated startle, women

## Abstract

Posttraumatic stress disorder (PTSD) is a common psychological disorder that affects a substantial minority of individuals. Previous research has suggested that PTSD can be partially explained as a disorder of impaired fear inhibition. The current study utilized a previously validated fear acquisition and extinction paradigm in a sample of 75 undergraduate women who were exposed to a campus mass shooting that occurred in 2008. We used a protocol in which conditioned fear was first acquired through the presentation of one colored shape (reinforced conditioned stimulus, CS+) that was paired with an aversive airblast to the larynx (unconditioned stimulus, US) and a different colored shape that was not paired with the airblast (non-reinforced conditioned stimulus, CS-). Fear was extinguished 10 min later through repeated presentations of the CSs without reinforcement. Number of clinically significant posttraumatic stress symptoms (PTSS) immediately following the mass shooting were positively associated with fear-potentiated startle (FPS) to the CS+ and CS- during late periods of acquisition. During early periods of fear extinction, PTSS was positively associated with FPS to the CS+. Results from the current study suggest that PTSS is related to altered fear inhibition and extinction during an FPS paradigm. In line with similar research, women with greater PTSS demonstrated a greater “fear load,” suggesting that these women experienced elevated fear to the CS+ during extinction after conditioned fear was acquired.

## Introduction

Posttraumatic stress disorder (PTSD) is a psychological disorder that affects approximately 8% of the United States population (Kessler et al., 2005). While men are more likely than women to report a history of potentially traumatic events (e.g., physical assault, combat, disaster/fire), women are frequently found to be at higher risk of developing PTSD (see for reviews, Tolin and Foa, 2006; Briscione et al., 2016). The characteristic symptoms of PTSD include persistent re-experiencing of the trauma (e.g., intrusive thoughts, distressing dreams), avoidance of stimuli associated with the trauma, negative changes in cognitions and mood, and increased arousal (e.g., difficulty falling/staying asleep, hypervigilance; American Psychiatric Association, 2013).

Utilizing the processes of fear conditioning (see Briscione et al., 2014, for a review of fear conditioning as a translational, experimental paradigm), extant literature has conceptualized PTSD as a disorder of impaired fear inhibition (Rothbaum and Davis, 2003; Amstadter et al., 2009; Norrholm et al., 2011). Specifically, during a potentially traumatic event, an individual’s sympathetic nervous system (SNS) response is typically activated (Amstadter et al., 2009). This SNS “fight-or-flight” response triggers sudden changes in the body, such as increased heart rate and blood pressure, the constriction of veins in order to send more blood to major muscle groups, and the shutting down of non-essential systems (e.g., immune system). The traumatic event serves as an unconditioned stimulus (US), while the “fight-or-flight” reaction serves as an unconditioned response (UR). This UR can be intimately paired with stimuli in the traumatic environment (e.g., smells, sounds); these stimuli subsequently become conditioned stimuli (CS). Therefore, an individual with PTSD experiences an UR (i.e., “fight-or-flight” response) to a CS that was formerly associated with the traumatic event, even when that individual is in the presence of safety (Davis, 1992). This response to the CS without the presence of the US demonstrates poor fear inhibition, or the inability to inhibit the fear response.

Recently, researchers have utilized fear conditioning paradigms in order to examine fear learning and inhibition in PTSD (Morgan et al., 1995; Jovanovic et al., 2005). Specifically, these paradigms measure fear-potentiated startle (FPS), which is defined as the relative increase in the acoustic startle response when a subject is presented with a CS (e.g., a colored shape) that is paired with an aversive US (e.g., an airblast to the larynx; Grillon and Morgan, 1999; Jovanovic et al., 2005, 2011). Given that the amygdala (a brain region implicated in the fear response) is directly connected to the neural circuitry of the startle reflex, the acoustic startle response is an effective measure of fear processing (Davis et al., 1993). Research has demonstrated that both civilian and veteran participants with PTSD exhibit greater FPS than those without PTSD (e.g., Grillon et al., 1998; Grillon and Morgan, 1999; Jovanovic et al., 2009; Norrholm et al., 2011; Sijbrandij et al., 2013). Furthermore, subjects with PTSD are unable to inhibit the fear response when they are presented with a safety signal (i.e., a colored shape that was not previously paired with an aversive airblast) during these paradigms (Davis et al., 1993; Norrholm et al., 2011; Sijbrandij et al., 2013). Essentially, this FPS paradigm has demonstrated that individuals with PTSD symptoms overgeneralize the US-UR association to multiple CSs; therefore, they cannot appropriately inhibit fear that they have paired with the threatening stimuli to the non-threatening stimuli.

These fear conditioning paradigms also measure fear extinction, which is conceptualized as a form of new learning that takes place when the previously reinforced CS is no longer paired with an aversive US (Jovanovic et al., 2009; Sotres-Bayon and Quirk, 2010). Norrholm et al. (2011) have demonstrated that traumatized subjects with PTSD (compared to traumatized subjects without PTSD) showed increased FPS responses to the previously reinforced CS during early and middle stages of extinction. This high level of FPS during early and middle stages of extinction has been termed “fear load” (see Norrholm et al., 2014), and it has been associated with longer extinction times (i.e., individuals with greater fear load take longer to extinguish than those with lower fear load; Norrholm et al., 2011).

The goal of the current study was to build upon previous research by examining FPS in a sample of women exposed to a campus mass shooting. On February 14, 2008, a gunman opened fire on the Northern Illinois University campus, killing five students and wounding 21 others. At the time of the shooting, 812 undergraduate women were enrolled in a longitudinal study and had provided extensive data on prior trauma history and posttraumatic stress symptoms (PTSS). Shortly after the shooting (approximately 17 days), those women were contacted and asked to complete questionnaires related to their reactions to the mass shooting. These women were assessed at numerous additional time points via a battery of online self-report measures following the campus mass shooting. Starting in May 2013, which was approximately 5 years post-shooting, these women were invited to participate in a FPS paradigm. Based on previous research, we hypothesized that women with higher PTSS immediately following the campus mass shooting would demonstrate greater FPS (assessed approximately 5 years post-shooting) to a safety signal and altered fear extinction during a FPS paradigm than women with lower PTSS immediately following the campus mass shooting. Extant literature has demonstrated that increased FPS (as well as deficits in extinction learning) represent an observable intermediate phenotype for risk for greater PTSS (Norrholm et al., 2014); therefore, the current study functioned under the assumption that this phenotype was stable.

## Materials and Methods

### Participants

Participants were undergraduate women who were initially recruited as part of a longitudinal study examining sexual revictimization. The only prerequisites for the longitudinal sexual revictimization study were that participants be women over the age of 18 and fluent in English. Trauma history was not a selection criterion. Following the campus mass shooting on February 14th, 2008, the Northern Illinois University (NIU) Trauma Study was launched in order to examine risk and resiliency factors after exposure to the mass shooting. See **Table 1** for a summary of participants’ exposure to the campus mass shooting. The women who participated in the initial longitudinal study on sexual revictimization were invited to participate in the NIU Trauma Study, as these women had already provided extensive data on previous trauma history and PTSS. Participants in the current study (*N* = 75) were recruited for the FPS paradigm via e-mails, telephone calls, and mass mailings. They were compensated 125.00 dollars for their participation. Prior to undergoing the FPS paradigm, all participants provided written informed consents. Current pregnancy, vision impairment, and hearing impairment were exclusion criteria. Seven women were excluded from the study due to current pregnancy. Most participants were in their Freshman year at the time of the shooting (*N* = 47, 63.5%).This study was approved by the NIU Institutional Review Board.

**Table 1:** Exposure to the campus mass shooting.

Variable	Yes *N* (%)	No *N* (%)
Were you on campus when the shooting occurred?	56 (74.7)	19 (25.3)
Did you see police or other personnel surrounding the buildings?	53 (70.7)	22 (29.3)
Did you see individuals who had been wounded or killed?	17 (22.7)	58 (77.3)
Do you know anyone that was wounded in the shooting?	21 (28.0)	54 (72.0)
Were you in a building that was placed on lockdown during the shooting?	34 (45.3)	41 (54.7)


### Self-Report Measures

The Distressing Events Questionnaire (DEQ; Kubany et al., 2000) was used to assess PTSS immediately following the campus mass shooting. The DEQ is a 17-item self-report measure that assesses the 17 symptoms of PTSD according to the *DSM-IV-TR* (American Psychiatric Association, 2000)^1^. Items are rated on a scale of 0 (*Absent or did not occur*) to 4 (*Present to an extreme or severe degree*). Participants were instructed to answer the items on the DEQ based on the shooting event. Research has demonstrated that a total score of 18 or above on the DEQ is indicative of “probable PTSD” among women (Kubany et al., 2000). Therefore, a cut-off score of 18 (and above) was used in the current study to assess “probable PTSD” (as a categorical variable) as a result of the campus mass shooting. In addition, we calculated PTSS as a count variable (i.e., count of the number of 17 items on the DEQ endorsed as “moderate” or “high”) in order to assess PTSD on a dimensional rather than categorical level (e.g., Orcutt et al., 2014).^2^ The DEQ has demonstrated strong psychometric properties, such as good test–retest reliability, good convergent and discriminant validity, and excellent internal consistency (Kubany et al., 2000). Cronbach’s alpha in the current study was 0.92.

The DEQ was also used to assess current PTSS. One week prior to undergoing the FPS paradigm, participants were emailed a link to an online survey. This online survey contained a battery of self-report questionnaires, including the DEQ. Participants were first given a brief trauma history screen [Traumatic Life Events Checklist (TLEQ); Kubany, 2004] in order to assess exposure to a range of potentially traumatic events. After participants completed the TLEQ, they were asked to denote which traumatic event was the most distressing (if they endorsed exposure to multiple potentially traumatic events). Participants subsequently responded to items on the DEQ according to the traumatic event that they endorsed as the most distressing. Therefore, participants’ current PTSS may have been as a result of the campus mass shooting, or their current symptoms may have been as a result of a different traumatic event. The average length of time between the DEQ assessed immediately after the shooting and the DEQ assessed before the FPS session was 286.88 weeks (*SD* = 13.35) or 5.52 years (*SD* = 0.26). Similar to the DEQ assessment immediately postshooting, a count of items endorsed on the DEQ as “moderate” or “high” was calculated for descriptive purposes.

### Psychophysiological Assessment

The startle reflex magnitude was measured using the electromyography (EMG) module of the BIOPAC MP150 for Windows (Biopac Systems, Inc., Aero Camino, CA, USA). The acquired data were filtered, rectified, and smoothed using the AcqKnowledge software suite (Biopac Systems, Inc., Aero Camino, CA, USA) and exported for statistical analyses. The EMG signal was sampled at a rate of 1000 Hz and filtered with low- and high-frequency cutoffs at 28 and 500 Hz, respectively. The maximum amplitude of the eye-blink muscle contraction 20–200 ms after presentation of the startle probe was used as a measure of the acoustic startle response.

The eye-blink component of the acoustic startle response was measured by EMG recordings of the right orbicularis oculi muscle with two 5-mm Ag/AgCl electrodes positioned 1 cm below the pupil of the right eye and 1 cm below the lateral canthus. Impedance levels were less than 6 kΩ for each participant. The startle probe was a 108-dB (A) SPL, 40-ms burst of broadband noise with near instantaneous rise time, delivered through headphones.

The FPS protocol consisted of two phases: Fear Acquisition and Fear Extinction. The Fear Acquisition phase consisted of three blocks with four trials of each type (CS+, CS-, and startle noise probe alone, NA) for a total of 12 trials per block and 36 total trials. Both CSs were colored shapes presented on a computer monitor for 6 s using SuperLab software (Cedrus, Inc.). At the end of this 6 s, startle probes were delivered for each trial type. The CS+ shape remained on the screen for another 0.5 after the startle probe ended and co-terminated with the airblast. The CS- shape terminated 0.25 s after the startle probe. The aversive US, a 250-ms airblast with an intensity of 140 p.s.i. directed at the larynx, was delivered 500 ms after the acoustic probe. This US has been used in several of our previous studies (e.g., Norrholm et al., 2011) and reliably produces robust FPS. In all phases, the inter-trial intervals were randomized to be 9–22 s in duration. Ten minutes after the Fear Acquisition phase, participants underwent the Fear Extinction phase. The Extinction phase consisted of six blocks with four trials of each type (the previously reinforced CS+, CS-, and NA) for a total of 12 trials per block and 72 total trials.

### Statistical Analysis

All statistical analyses were performed in IBM SPSS Statistics 21.0 for Windows, with α = 0.05. A one-way analysis of variance (ANOVA) was used to compare demographic information (e.g., age) and information on participants’ PTSD symptoms between the PTSD+ (i.e., participants who had a total score of 18 or greater on the DEQ) and PTSD- (i.e., participants who had a total score of less than 18 on the DEQ) groups. A chi-square analysis was used to compare categorical data (e.g., race). FPS was calculated using a difference score ([startle magnitude in the presence of a CS in each conditioning block] – [startle magnitude to the noise probe alone (NA)]). These variables were analyzed in a mixed ANOVA with the within-subject factors of Block (three levels for Acquisition; three levels for Extinction) and trial type (two levels, CS+ and CS-), and the between-groups factor of PTSD symptomatology (two levels, PTSD+ or PTSD-). Late Acquisition was defined as block 3 of Acquisition, when discrimination learning was at maximum. Extinction was divided into three phases: early (blocks 1 and 2), mid (blocks 3 and 4), and late (blocks 5 and 6). Furthermore, given the dimensional scale used for PTSS, we computed bivariate correlations in order to assess the relationship between PTSS and FPS to the CS+ and CS- during late Acquisition and all Extinction phases. Baseline startle was measured by comparing average startle magnitude to the noise probe alone between PTSD groups.

## Results

Seventy-five female participants underwent the FPS paradigm. Of those 75 women, 42 met criteria for probable PTSD according to the DEQ (PTSD+) immediately following the campus mass shooting, whereas 33 did not meet criteria for probable PTSD according to the DEQ (PTSD-). Of the 42 female participants who met criteria for probable PTSD immediately following the shooting, five participants met criteria for current probable PTSD 5 years later (assessed 1 week prior to their FPS paradigm session). Of the 33 female participants who did not meet criteria for probable PTSD following the shooting, one participant met criteria for current probable PTSD. **Table 2** provides detailed demographic information for the PTSD+ and PTSD- participants, as well as information on both groups’ reported PTSD symptomatology immediately following the mass shooting. The PTSD+ participants had higher total DEQ scores assessed immediately following the mass shooting event (expressed as a count variable) than the PTSD- participants, *F*(1,74) = 164.64, *p* < 0.001. Additionally, the PTSD+ participants had higher symptom cluster count scores for re-experiencing [*F*(1,74) = 72.33, *p* < 0.001], avoidance [*F*(1,74) = 79.35, *p* < 0.001], and hyperarousal [*F*(1,74) = 89.51, *p* < 0.001] than the PTSD- participants. With regard to current PTSS, the PTSD+ participants had significantly higher total count scores than PTSD- participants [*F*(1,70) = 6.67, *p* < 0.05], as well as significantly higher count scores for avoidance and hyperarousal (but not re-experiencing).

**Table 2:** Participantdemographic and posttraumatic stress disorder (PTSD) symptomatology data.

Demographics	PTSD+ (*n* = 42)	PTSD- (*n* = 33)	
Race (% White)	83.3	71.9	*p* > 0.05
Current age (*M*, *SD*)	25.18 (1.57)	25.72 (2.75)	*p* > 0.05
PTSD Symptoms immediately postshooting	*M* (*SD*) (n = 42)	*M* (*SD*) (*n* = 33)	
Total	10.62 (3.09)	2.55 (2.11)	*F*(1,74) = 164.64^∗∗^
Re-experiencing	3.38 (1.50)	0.79 (1.02)	*F*(1,74) = 72.33^∗∗^
Avoidance	3.71 (1.73)	0.67 (1.05)	*F*(1,74) = 79.35^∗∗^
Hyperarousal	3.52 (1.11)	1.09 (1.10)	*F*(1,74) = 89.51^∗∗^
PTSD Symptoms prior to FPS session	*M* (*SD*) (*n* = 40)	*M* (*SD*) (*n* = 31)	
Total	2.58 (3.23)	0.97 (1.43)	*F*(1,70) = 6.67^∗^
Re-experiencing	0.88 (1.30)	0.35 (0.84)	*F*(1,70) = 3.73
Avoidance	0.83 (1.20)	0.23 (0.50)	*F*(1,70) = 6.85^∗^
Hyperarousal	1.02 (1.23)	0.45 (0.81)	*F*(1,70) = 5.04^∗^


*PTSD symptoms were modeled as a count variable (count of the number of 17 items on the DEQ endorsed as “moderate” or “high”). PTSD+ = “probable PTSD”; PTSD- = “no probable PTSD.” ^∗^*p* < 0.05, ^∗∗^*p* < 0.01.*

### Conditioned Fear Acquisition: Fear-Potentiated Startle

**FIGURE 1:**
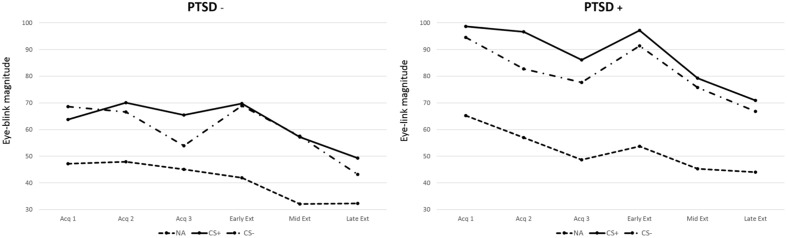
**Eye-blink magnitude for noise alone (NA), CS+, and CS- across three acquisition blocks and three extinction blocks (early, mid, and late) by posttraumatic stress disorder (PTSD) status (present/absent)**.

There was no Group difference in acoustic startle to noise alone (NA) trials between the PTSD+ and PTSD- groups during Fear Acquisition [*F*(1,74) = 1.28, *p* > 0.05], indicating that baseline startle was equal across all participants. A repeated-measures ANOVA of FPS during the late Acquisition phase (defined as the third block of the Acquisition phase) with trial type (CS+, CS-) as a within-subjects variable and PTSD symptomatology (PTSD+, PTSD-) as a between-subjects variable revealed a significant main effect of trial type [*F*(1,73) = 7.37, *p* = 0.008]. The interaction between trial type and PTSD symptomatology group was non-significant [*F*(1,73) = 0.29, *p* > 0.05]. During late Acquisition, participants demonstrated robust FPS to the CS+ compared to the NA trials [*F*(1,73) = 12.13, *p* = 0.001]; there was no Group difference between the PTSD+ and PTSD- subjects (see **Figure 1**).^3^

In order to assess whether altered fear inhibition is persistent in participants without current probable PTSD, we conducted a second mixed ANOVA and excluded the six participants with current probable PTSD (assessed 1 week prior to their FPS session). The Group difference between the PTSD+ and PTSD- subjects was approaching significance (*p* = 0.067); however, the interaction between trial type and PTSD symptomatology group was still non-significant [*F*(1,63) = 0.57, *p* > 0.05].

### Within-Session Fear Extinction: Fear-Potentiated Startle

There was no Group difference in baseline acoustic startle between the PTSD+ and PTSD- groups during Fear Extinction [*F*(1,73) = 1.12, *p* > 0.05]. Participants demonstrated robust within-session extinction of FPS to the previously reinforced CS+ [*F*(1,72) = 4.48, *p* = 0.001, main effect of block]; there was no Group difference between the PTSD+ and PTSD- subjects (see **Figure 1**).

Similar to analyses for fear acquisition, in order to assess whether impaired fear extinction is persistent in participants without current probable PTSD, we conducted another mixed ANOVA and excluded the six participants with current probable PTSD. The Group difference between the PTSD+ and PTSD- subjects was still non-significant, as well as the interaction between trial type and PTSD symptomatology group [*F*(1,59) = 0.81, *p* > 0.05].

### Analyses with PTSS as a Continuous (versus Categorical) Variable

As mentioned previously, the current study also calculated PTSS as a count variable (i.e., count of the number of 17 items on the DEQ endorsed as “moderate” or “high”) in order to assess PTSS on a dimensional rather than categorical level. Below are the results from our dimensional analyses.

**FIGURE 2:**
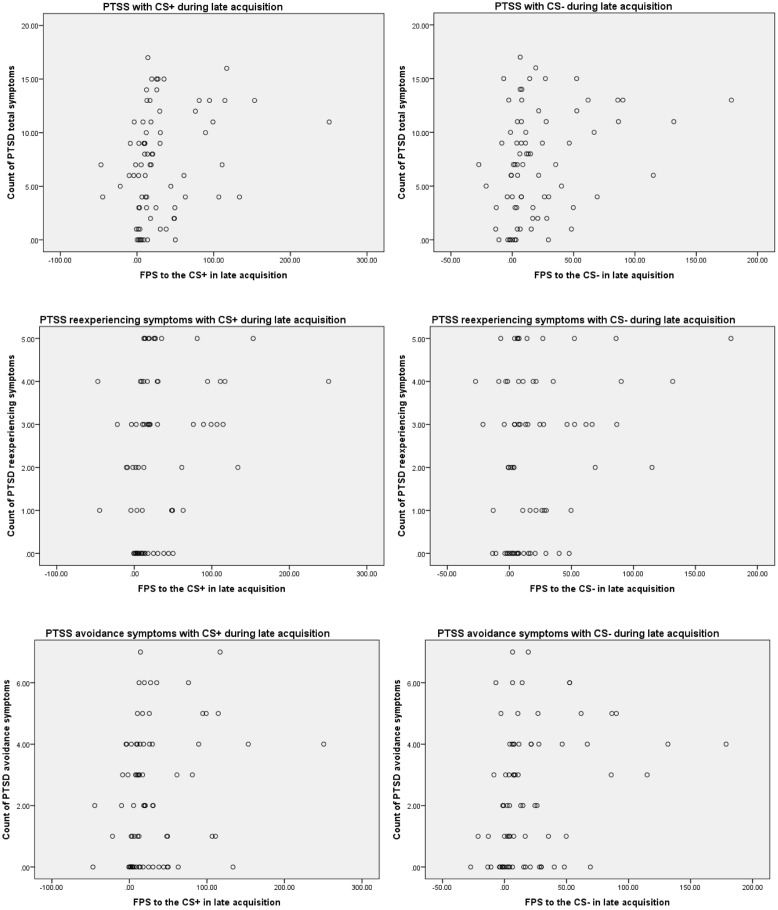
**Scatterplots depicting significant correlations among PTSS symptoms and fear-potentiated startle (FPS) to the CS+ and CS- in late acquisition**.

Posttraumatic stress symptoms was positively associated with FPS to the CS+ during late Acquisition (*r* = 0.276, *p* = 0.016) and with FPS to the CS- during late Acquisition (*r* = 0.292, *p* = 0.011). These findings suggest that participants with greater PTSS immediately following the campus mass shooting demonstrated greater FPS to both the CS+ and CS- during late Acquisition compared to participants with lower PTSS immediately following the campus mass shooting. In regard to specific PTSS clusters, the frequency of re-experiencing symptoms was positively associated with FPS to the CS+ during late Acquisition (*r* = 0.280, *p* = 0.013) and with FPS to the CS- during late Acquisition (*r* = 0.244, *p* = 0.035). Furthermore, the frequency of avoidance symptoms was positively associated with FPS to the CS+ during late Acquisition (*r* = 0.233, *p* = 0.044) and with FPS to the CS- during late Acquisition (*r* = 0.282, *p* = 0.014) (see **Figure 2** for acquisition scatterplots).

In order to assess whether these findings remain among participants without current probable PTSD, we conducted additional correlations excluding the six participants with current probable PTSD. PTSS (measured dimensionally) was still positively associated with FPS to the CS+ during late Acquisition (*r* = 0.295, *p* = 0.019) and with FPS to the CS- during late Acquisition (*r* = 0.348, *p* = 0.005). Furthermore, the frequency of re-experiencing symptoms was still positively associated with FPS to the CS+ during late Acquisition (*r* = 0.325, *p* = 0.009) and with FPS to the CS- during late Acquisition (*r* = 0.316, *p* = 0.012). However, the frequency of avoidance symptoms was positively associated with FPS to the CS- only during late Acquisition (*r* = 0.318, *p* = 0.011). In addition, the frequency of hyperarousal symptoms was positively associated with FPS to the CS- during late Acquisition (*r* = 0.262, *p* = 0.038).

**FIGURE 3:**
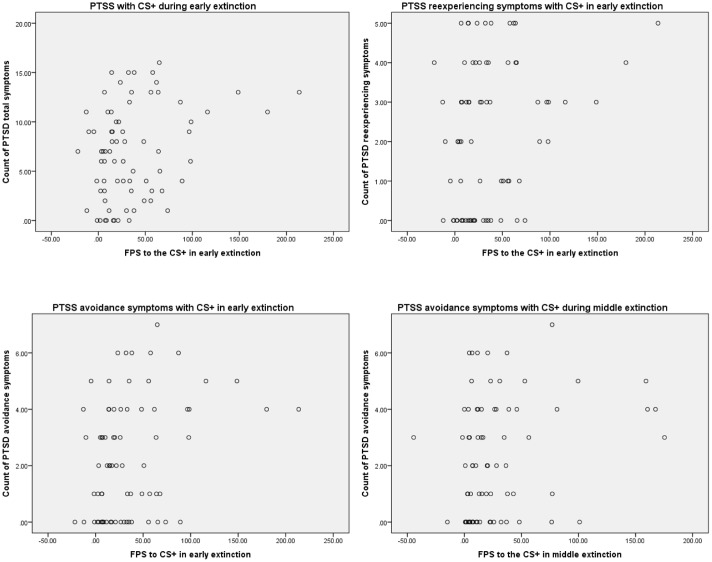
**Scatterplots depicting significant correlations among PTSS symptoms and FPS to the CS+ during extinction**.

During Extinction, PTSS was positively associated with FPS to the CS+ during early Extinction (*r* = 0.304, *p* = 0.009). This suggests that participants with greater PTSS demonstrated greater FPS to the CS+ during early Extinction compared to those with lower PTSS. Overall, these results suggest that participants with greater PTSS immediately following the campus mass shooting demonstrated a greater “fear load” than those with lower PTSS immediately following the campus mass shooting. In regard to specific PTSS clusters, the frequency of re-experiencing symptoms was positively associated with FPS to the CS+ during early Extinction (*r* = 0.267, *p* = 0.021). Furthermore, the frequency of avoidance symptoms was positively associated with FPS to the CS+ during early Extinction (*r* = 0.319, *p* = 0.006) and with FPS to the CS+ during mid Extinction (*r* = 0.243, *p* = 0.037). No individual PTSS clusters were correlated with FPS to the CS- during any stages of Extinction (see **Figure 3** for extinction scatterplots).

In order to assess whether these findings remain among participants without current probable PTSD, we conducted additional correlations excluding the six participants with current probable PTSD. PTSS (measured dimensionally) was still only associated with FPS to the CS+ during early Extinction (*r* = 0.308, *p* = 0.015), and the relationship between PTSS and FPS to the CS- during early Extinction was approaching significance (*r* = 0.236, *p* = 0.065). Furthermore, the frequency of re-experiencing symptoms was positively associated with FPS to the CS+ during early Extinction (*r* = 0.285, *p* = 0.024). In addition, the frequency of avoidance symptoms was positively associated with FPS to the CS+ during early Extinction (*r* = 0.313, *p* = 0.013); the relationship between avoidance symptoms and FPS to the CS+ during mid Extinction was approaching significance (*r* = 0.244, *p* = 0.056). Lastly, the relationship between avoidance symptoms and FPS to the CS- during early Extinction was also approaching significance (*r* = 0.238, *p* = 0.063).

## Discussion

Using a previously validated FPS paradigm, the current study examined FPS and PTSS in a sample of women exposed to a campus mass shooting. Specifically, it was hypothesized that women with probable PTSD immediately following the campus mass shooting would exhibit larger FPS responses (assessed approximately 5 years post-shooting) to a safety signal and altered fear extinction in comparison to those without probable PTSD. During fear acquisition, women with and without probable PTSD did not differ in their baseline startle responses. Previous research has also failed to find a difference in baseline startle between participants with and without PTSD (e.g., Jovanovic et al., 2010; Norrholm et al., 2011). Further, these groups did not differ in their FPS responses during late Acquisition. However, when examining PTSS as a continuous variable, women with greater PTSS demonstrated greater FPS responses to both the CS+ and CS- during late Acquisition. These results persisted even after removing all participants with current probable PTSD from the analyses. Therefore, high levels of PTSS appear to be associated with larger FPS responses during fear acquisition; however, this effect was not large enough in the current study to produce a significant between-subjects difference in the PTSD+ and PTSD- groups. After examining specific PTSS clusters, women with more re-experiencing and avoidance symptoms showed greater levels of fear to the CS+ and CS- during late Acquisition, compared to women with less re-experiencing and avoidance symptoms. These correlations persisted even after removing all participants with current probable PTSD from the analyses; in addition, the frequency of hyperarousal symptoms became positively associated with FPS to the CS- during late Acquisition.

Similar to the findings during fear acquisition, women with and without probable PTSD did not differ in their baseline startle responses during fear extinction. Furthermore, when PTSS was modeled continuously, greater PTSS was associated with greater FPS responses to the CS+ during early Extinction; these results also persisted after moving participants with current probable PTSD from the analyses. Although the ANOVA analyses did not suggest between-subject differences between the categorical PTSD+ and PTSD- groups in regard to FPS responses, these findings do suggest that PTSS measured continuously is significantly related to the acquisition and extinction of fear in the current study. The finding that greater PTSS was associated with greater FPS responses to the CS+ during early Extinction supports the notion that individuals with a greater number of clinically significant PTSD symptoms at time of trauma (i.e., the campus mass shooting) are more likely to have greater fear load that persists for years after trauma (e.g., Norrholm et al., 2011), even in the absence of current symptoms. After examining specific PTSS clusters, women with more re-experiencing and avoidance symptoms showed greater levels of fear to the CS+ during early Extinction, compared to women with less re-experiencing and avoidance symptoms. Furthermore, women with more avoidance symptoms showed greater levels of fear to the CS+ during middle Extinction, compared to women with less avoidance symptoms. These results also persisted after excluding participants with current probable PTSD from the analyses.

In regard to the findings related to specific PTSS clusters, it is interesting that most of the findings did not demonstrate a significant relationship between hyperarousal symptoms and impaired fear inhibition during acquisition or impaired fear extinction. The only significant finding was a positive correlation between hyperarousal symptoms and FPS to the CS- during late acquisition, after excluding participants with current probable PTSD. As classified in the *DSM-IV-TR* and the *DSM-5*, the exaggerated startle response symptom is listed as one of the hyperarousal symptoms. However, as noted by Jovanovic et al. (2009), the hyperarousal symptom cluster is more closely related to problems sleeping and sustaining focus, while the re-experiencing symptom cluster is more closely related to the inability to inhibit or control physiological arousal in response to trauma reminders. Therefore, it seems appropriate that impaired fear inhibition was more strongly related to re-experiencing symptoms than hyperarousal symptoms in the current study; this pattern of results has also been found in previous research (see Jovanovic et al., 2009).

A possible explanation for the lack of significant between-subjects differences among the PTSD+ and PTSD- groups may be that PTSD symptomatology naturally decreased from immediately following the campus mass shooting to the time that participants engaged in the FPS paradigm. For example, of the 42 female participants who met criteria for probable PTSD immediately following the shooting, only five participants met criteria for current probable PTSD. Given that many women in the NTS study experienced a PTSD trajectory of resilience (see Orcutt et al., 2014), it is possible that current low levels of PTSS helps to explain the lack of significant between-subjects findings. However, it is still notable that when PTSS (assessed immediately after the campus mass shooting) were measured continuously, women with greater PTSS demonstrated impaired fear inhibition and a greater fear load than those with lower PTSS, even after excluding those with current probable PTSD from the analyses.

The primary finding that continuous symptom, but not diagnostic category, analyses were associated with FPS is consistent with the National Institute of Mental Health (NIMH) Research Domain Criteria framework, in which neuroscience based, dimensional approaches are advocated in the study of mental disorders. The use of the startle reflex provides an observable, biologically based metric of vulnerability. Although the study did not assess FPS prior to the shooting, the fact that heightened fear responses are seen 5 years later suggests that fear load may either be (1) a pre-existing risk factor that in the immediate aftermath of the trauma increased the frequency of symptoms, or (2) a long-term consequence of greater PTSS. Future studies of fear load using a prospective approach could tease apart these two hypotheses.

An additional limitation was relying only upon a self-report measure to assess PTSS, which may be less accurate than those obtained via clinical interview. It is also important to consider how the sample recruitment may contribute to bias in the findings. In the present study, participants were originally recruited into the longitudinal study through an Introductory Psychology research pool and received course credit. Following the mass shooting, participants who had agreed to be recontacted and were still enrolled as students were invited to complete the 30-min online survey with the option of receiving $40. A large percentage were recruited into the ongoing longitudinal study (approximately 85%). The females in the present study were drawn from this group and were offered $125 to travel to NIU and participate in the 2-h study. Anecdotally, many participants expressed that participating in the study was a way to contribute to something positive after the mass shooting. The circumstances around the shooting and the financial compensation may have introduced unknown bias into the findings. In addition, the sample from the current study only consisted of female undergraduate students, thus reducing generalizability to other populations. However, a notable strength of the current study is that it utilized a sample of women exposed to a homogenous trauma – a campus mass shooting.

Despite these limitations, results from the current study may have important clinical implications. The current study found that women with greater PTSS following the mass shooting exhibited an especially strong fear load during extinction. This finding highlights the importance of treatments focused on the facilitation of extinction learning, particularly given that extinction learning can be viewed as a laboratory analog of exposure therapy (Rothbaum and Davis, 2003). Previous research has found that engagement in exposure-based psychotherapies, such as Prolonged Exposure (PE; Foa et al., 2007), results in significant reductions in PTSD symptoms. Psychopharmacology researchers have begun to pair exposure-based interventions (such as PE) with pharmacological means [such as d-cycloserine (DCS; an antibiotic that may enhance learning and memory)] to assess whether the combination of these treatments leads to a greater increase in extinction learning (Walker et al., 2002). While research findings have been mixed regarding the effectiveness of DCS in facilitating extinction learning in PTSD, Difede et al. (2014) recently found greater PTSD symptom reduction and remission rates among individuals who were administered DCS prior to virtual reality exposure therapy compared to those who were administered a placebo prior to the therapy. Additional research is needed, however, to more fully understand the conditions in which DCS, as well as other pharmacological means, is most effective in enhancing extinction learning in those with PTSD.

## Conclusion

The results from the current study are generally consistent with previous research suggesting that greater PTSS immediately following exposure to a traumatic event are related to altered fear inhibition and extinction during an FPS paradigm, even in the absence of current PTSS. In line with similar research (e.g., Norrholm et al., 2011; Fani et al., 2012; Glover et al., 2013; Inslicht et al., 2013), women with greater PTSS immediately following the mass shooting demonstrated a greater fear load, suggesting that these women experienced elevated fear to the CS+ during fear extinction after conditioned fear was acquired. This elevated fear to the CS+ was most prominent during the early stages of Extinction, suggesting that methods for reducing fear or anxiety during extinction-based exposure psychotherapies may prove valuable in treating PTSD (Davis et al., 2006).

## Ethics Statement

This study was carried out in accordance with the recommendations of Institutional Review Board at Northern Illinois University with written informed consent from all subjects. All subjects gave written informed consent in accordance with the Declaration of Helsinki. The protocol was approved by the Institutional Review Board at Northern Illinois University.

## Author Contributions

HO, SH, AS, TJ, SN, KR, and TM have made substantial, direct, and intellectual contribution to the work and have approved it for publication.

## Conflict of Interest Statement

The authors declare that the research was conducted in the absence of any commercial or financial relationships that could be construed as a potential conflict of interest.

## Footnotes

1 PTSD criteria for DSM-5 were not yet published at the time the current study began data collection.

2 To examine the long term impact of the shooting, we wanted to capture the number (or count) of PTSD symptoms that people were experiencing years after the shooting that were at least moderately distressing. We wanted to be able to make a distinction between participants who may still be endorsing several symptoms at a very mild level versus a participant who was endorsing fewer symptoms, but was reporting these symptoms as more distressing.

3 Analyses were also conducted with a median split of PTSD symptoms (i.e., high vs. low PTSD symptoms) as the between-groups factor in a mixed ANOVA, with the within-subject factors of Block (three levels for Acquisition; six levels for Extinction) and trial type (two levels, CS+ and CS-). Results were comparable and the interaction between trial type and PTSD symptoms (high vs. low) was also non-significant.
